# NEIGHBORHOOD GREENSPACE AND GRAY MATTER MICROSTRUCTURAL INTEGRITY: THE HEALTH ABC STUDY

**DOI:** 10.1093/geroni/igac059.025

**Published:** 2022-12-20

**Authors:** Sara Godina, C Shaaban, Caterina Rosano, Howard Aizenstein, Andrea Rosso

**Affiliations:** University of Pittsburgh School of Public Health, Pittsburgh, Pennsylvania, United States; Department of Epidemiology, University of Pittsburgh School of Public Health, Pittsburgh, Pennsylvania, United States; University of Pittsburgh, Pittsburgh, Pennsylvania, United States; Department of Psychiatry, University of Pittsburgh School of Medicine, Pittsburgh, Pennsylvania, United States; University of Pittsburgh, Pittsburgh, Pennsylvania, United States

## Abstract

Neighborhood greenspace is positively associated with cognition and macrostructural indicators of brain health. Microstructural damage may be a sensitive marker for dementia-related neurodegeneration that precedes such macrostructural changes. We aimed to examine cross-sectional associations between neighborhood (census tract) greenspace and gray matter (GM) microstructure in 265 community-sampled adults (mean age=83, 57% women, 39% Black). Linear mixed effects regression models tested associations between quartiles of percent greenspace derived from the normalized vegetation index and mean diffusivity (MD) quantified using magnetic resonance imaging with diffusion tensor. Greater greenspace was related to higher MD in 4 regions: left calcarine (Q3 vs. Q1 stβ=0.38, p=0.0348), left thalamus (Q2 vs. Q1 stβ=0.35, p=0.0443; Q3 vs. Q1 stβ=0.49, p=0.0061), and bi-lateral precuneus (left Q2 vs. Q1 stβ=0.46, p=0.0096; right Q2 vs. Q1 stβ=0.36, p=0.0385). The relationship between greenspace and cognition may be through paths other than GM microstructure; future research should explore other potential mechanisms.

